# EvolvingSTEM: a microbial evolution-in-action curriculum that enhances learning of evolutionary biology and biotechnology

**DOI:** 10.1186/s12052-019-0103-4

**Published:** 2019-04-24

**Authors:** Vaughn S. Cooper, Taylor M. Warren, Abigail M. Matela, Michael Handwork, Shani Scarponi

**Affiliations:** 1 Department of Microbiology and Molecular Genetics, University of Pittsburgh, School of Medicine, Pittsburgh, PA USA; 2 Center for Evolutionary Biology and Medicine, University of Pittsburgh, School of Medicine, Pittsburgh, PA USA; 3 Department of Molecular, Cellular, and Biomedical Sciences, University of New Hampshire, Durham, NH USA; 4 Winnacunnet High School, Hampton, NH USA

## Abstract

Evolution is a central, unifying theory for all of life science, yet the subject is poorly represented in most secondary-school biology courses, especially in the United States. One challenge to learning evolution is that it is taught as a conceptual, retrospective subject with few tangible outcomes for students. These typical passive learning strategies lead to student disengagement with the material and misunderstanding of evolutionary concepts. To promote greater investment and comprehension, we developed EvolvingSTEM, an inquiry-based laboratory curriculum that demonstrates concepts of natural selection, heredity, and ecological diversity through experimental evolution of a benign bacterium. Students transfer populations of *Pseudomonas fluorescens* growing on plastic beads, which selects for biofilm formation and mutants with new, conspicuous phenotypes. We introduced our curriculum to four introductory high school biology classes alongside their standard curriculum materials and found that students who learned evolution through EvolvingSTEM scored significantly better on a common assessment targeted to Next Generation Science Standards than students taught only the standard curriculum. This latter group subsequently achieved similar scores once they too completed our curriculum. Our work demonstrates that inquiry-based, hands-on experiences with evolving bacterial populations can greatly enhance student learning of evolutionary concepts.

**Electronic supplementary material:**

The online version of this article (10.1186/s12052-019-0103-4) contains supplementary material, which is available to authorized users.

## Introduction

Understanding evolutionary processes is fundamental to all areas of life science because evolution serves as a conceptual framework to organize other life science topics, such as organismal diversity and ecological interactions. Furthermore, some of the most significant threats to human health are evolutionary phenomena; therefore, knowledge of evolutionary processes has a direct impact on public health and medicine (Wells et al. 2017). For example, antimicrobial resistance and cancer are caused by the rapid evolution of microbes and our own cells, respectively (Karatan and Watnick 2009; Greaves and Maley 2012; Berendonk et al. 2015; Makohon-Moore and Iacobuzio-Donahue 2016; Alizon and Méthot 2018). In addition, ongoing revolutions in biotechnology and personalized medicine, such as gene-editing (i.e., CRISPR), can only be understood in the context of the evolutionary concept of descent from a shared ancestral lineage (Makarova et al. 2015; Knott and Doudna 2018). A strong knowledge base of evolution is therefore invaluable for a literate society to understand scientific and medical advances and for a prepared workforce to excel in jobs in science, technology, and engineering. The value of evolutionary biology knowledge is highlighted by its inclusion as a core concept for STEM education practices (National Research Council 2012; NGSS Lead States 2013; NSTA 2013).

Although the importance of evolutionary biology is well-established, misconceptions of its basic principles remain prevalent among students, the general public, and even the teachers who are providing instruction (Cunningham and Wescott 2009; Gregory 2009; Sickel and Friedrichsen 2013; Yates and Marek 2014; Glaze and Goldston 2015). While many concurrent factors likely contribute to poor understanding (Smith 2010a, b; Pobiner 2016), one potential reason that evolutionary concepts are misunderstood is that typical curricula use passive learning strategies, where instruction relies on lectures and textbook readings. Current evolution curriculum design runs counter to evidence that student-centered, active learning strategies are the most effective method for science teaching and have been shown to improve student understanding of evolutionary concepts (Nehm and Reilly 2007; Nelson 2008; Freeman et al. 2014; Romine et al. 2017). Courses that provide students with authentic research experiences are especially effective at increasing student engagement and promoting a deeper understanding of evolution (Jordan et al. 2014; Ratcliff et al. 2014; Broder et al. 2018).

There is therefore a critical need for engaging and informative evolutionary biology curricula that provide K-12 students the opportunity to explore the concept of changing frequencies of inherited traits just as they attempt to quantify gravity in physics or acid–base reactions in chemistry. To meet this need, we developed EvolvingSTEM, a curriculum that provides inquiry-based learning of evolution, microbiology, ecology, and heredity with a laboratory experiment that employs real scientific research practices. EvolvingSTEM allows students to visualize evolutionary adaptations arising in real time by growing populations of the harmless bacterium *Pseudomonas fluorescens* under conditions that select for the formation of a biofilm. A biofilm is a surface dwelling community of microbes encased in a protective coating of self-produced polymers; biofilms are the dominant form of microbial life (Costerton et al. 1987). They are also structured, heterogeneous environments that include varied ecological niches (Karatan and Watnick 2009). Bacteria with advantageous mutations colonize these niches, and their adaptations cause visible differences in colony morphology from the ancestral genotype (Rainey and Travisano 1998; Flynn et al. 2016). This evolution-in-action occurs within days, requires little specialized equipment, and can be offered in any classroom laboratory that can support sterile technique. Our curriculum is intended to replace standard, passive learning curricula to meet competencies for natural selection and evolution described in the Next-Generation Science Standards (HS-LS4, (NGSS Lead States 2013)). We hypothesized that students who learn evolutionary concepts with our curriculum would have significant increases in content knowledge relative to students that were provided only the standard curriculum.

## Results

### Developing and refining an amenable protocol for teaching bacterial evolution to high school students

The idea to teach evolutionary concepts to high school students with a bacterial evolution experiment grew from our research on identifying the causes of rapidly evolving mutant colony morphologies of the opportunistic pathogens *Burkholderia cenocepacia* and *Pseudomonas aeruginosa* (Poltak and Cooper 2011; Flynn et al. 2016). These species are particularly threatening to persons with cystic fibrosis, where they cause chronic airway infections by forming biofilms (Starkey et al. 2009; Ashish et al. 2013). Biofilm-associated infections are inherently more resistant to host immunity and antimicrobials because secreted adhesive polymers are protective and the cells within grow more slowly (Harrison et al. 2005). Eventually, some bacteria disperse from the colony, either as individuals or clusters, to inhabit new surfaces and resume the biofilm lifecycle (Poltak and Cooper 2011; Martin et al. 2016).

**Fig. 1:**
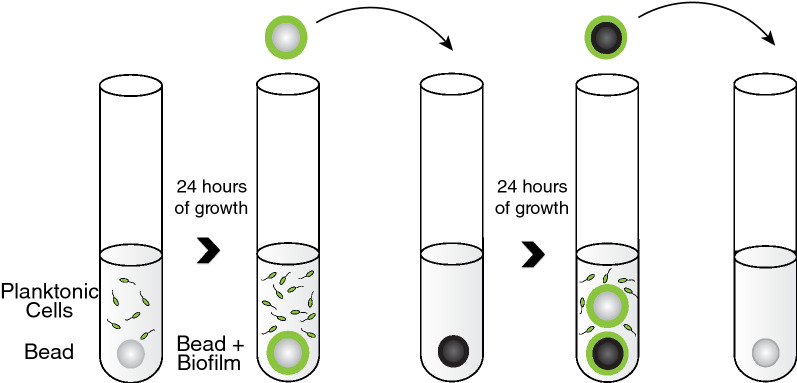
Biofilm lifecycle model. Bacteria are grown in test tubes with plastic beads on which biofilm forms. Daily bead transfers select for bacterial attachment, assembly, dispersal, and reattachment (figure adapted from Turner et al. 2018)

In order to study the dynamics of bacterial evolution in vitro, we developed a simple method to model the biofilm lifecycle of surface attachment, biofilm formation, dispersal, and recolonization (Fig. 1, Poltak and Cooper 2011; Traverse et al. 2013; O’Rourke et al. 2015; Flynn et al. 2016; Turner et al. 2018). In short, we culture bacteria for 24 h in test tubes containing growth media and a polystyrene bead. A subset of the bacteria colonize the bead and form a biofilm. We then transfer only the biofilm-covered bead to a new tube with a fresh bead. We repeat this process daily to select for bacterial mutants that are best adapted to aspects of the entire biofilm lifecycle. Conveniently, we found that biofilm adapted mutants also display altered colony morphologies when grown on agar plates, making them conspicuous to students.

In collaboration with science teachers and administrators at Winnacunnet High School (Hampton, NH, USA), we modified our research laboratory protocol to accommodate implementation in a high school classroom. We selected the plant probiotic bacterium, *Pseudomonas fluorescens* SBW25, as our study subject because it had several qualities that made it a good candidate for use in a high school classroom: (1) it is benign, and thus safe for students with no microbiology experience, (2) it had previously been suggested as a good candidate for use in educational settings (Green et al. 2011; Spiers 2014), and (3) it is the subject of a large body of research on its capacity for rapid and conspicuous adaptive evolution in biofilm-related conditions (Rainey and Travisano 1998; Spiers 2005). Adaptive *P. fluorescens* mutants are often characterized by rugose or rosette-like colony morphologies resulting from greater production of polysaccharides for attachment (Rainey et al. 2000). We found that experimental evolution of *P. fluorescens* SBW25 in the biofilm lifecycle model selected for a high frequency of adaptive mutants with novel colony morphologies in less than 2 weeks.

To accelerate this process and ensure that our experiment could be performed within the timeframe of a high school biology lesson, we conducted a series of trials in different media to determine conditions that resulted in predictable, rapid adaptations. We found that growth in King’s B medium (KB) generated multiple, heritable colony phenotypes within 7 days. In the interest of accelerating the evolutionary dynamics, we repeated the experiment in KB medium with various glycerol concentrations. We found that an increase from 1.5 to 2.5% glycerol selected for novel colony morphologies at detectable frequencies in 4 days. We named this modified media recipe “Queen’s B” (QB) and used this recipe thereafter. Media recipes are available in Additional file 1.

**Fig. 2:**
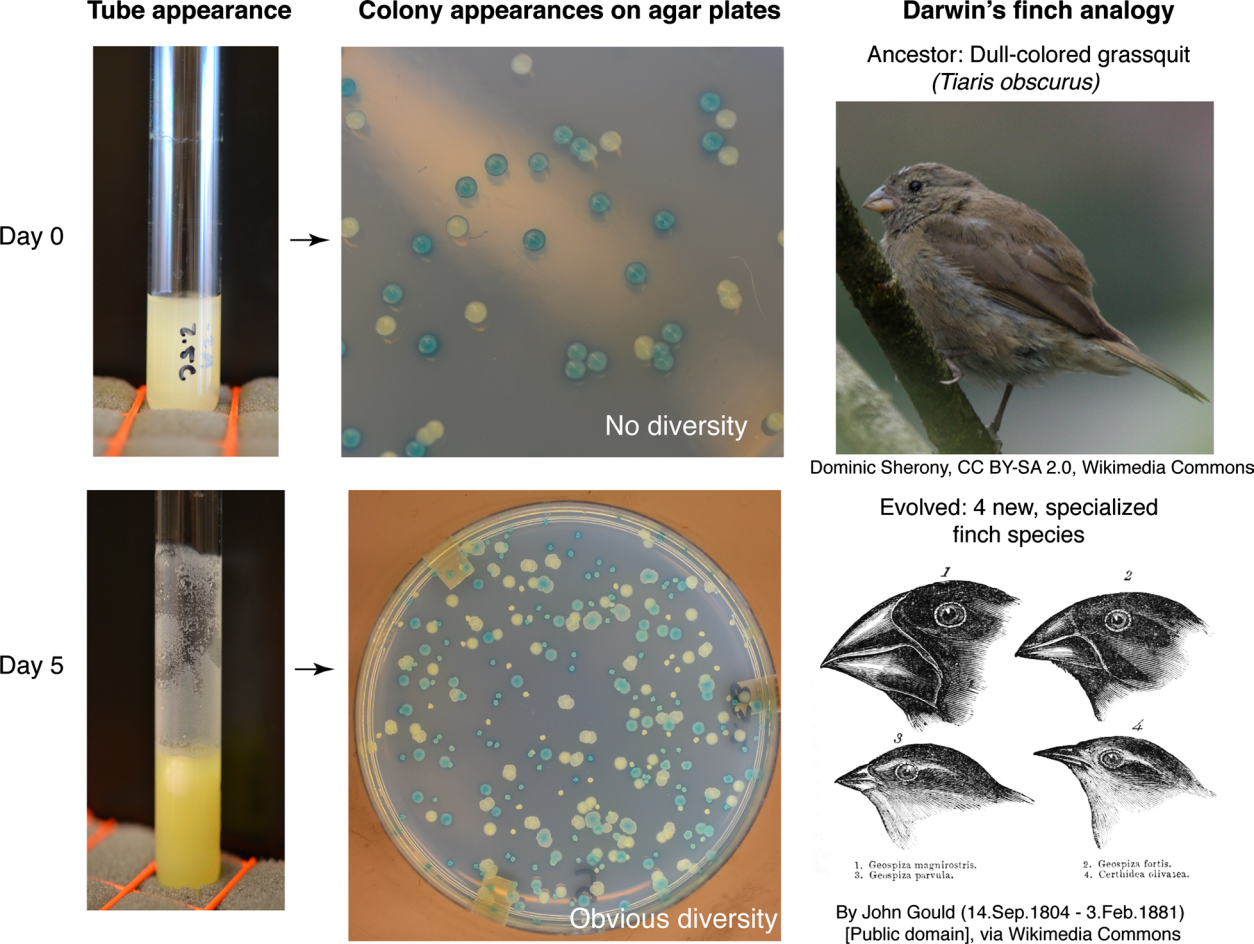
Adaptation to biofilm selection can occur within days and produce conspicuous phenotypic differences. Populations were founded with equal ratios of Lac + (blue) and Lac- (white) ancestral genotypes that do not differ in morphology. After 5–7 days, new colony morphologies evolve and represent different biofilm-associated ecological strategies, as different beak shapes of Darwin’s finches represent distinct feeding strategies (Rainey and Travisano 1998; Poltak and Cooper 2011)

Students can use our modified protocol to guide an inquiry-based experiment that allows them to visualize evolution in their bacterial populations in only six class periods (Fig. 2). For example, on Monday, students inoculate glass test tubes containing QB media and a polystyrene bead with a clone of *P. fluorescens* SBW25, and then perform bead transfers for the following 3 days (Tuesday–Thursday). During the process of bead transfer, students can identify effects of natural selection by observing increased biofilm production on the walls of their test tubes. In addition, at the beginning and end of the week, students sample their populations by growing individual bacterial colonies on agar plates. Students can make observations of mutant colonies on the Monday of the following week and compare these colonies to those of the ancestral population that were plated earlier in the week. Students can be given additional curriculum materials, such as homework and pretests, to prepare them for each step in the laboratory protocol and provide opportunities for them to link the heritable, adaptive evolutionary change they observe in their experiment to the evolutionary processes that produced this dynamic. Through EvolvingSTEM, students can acquire the knowledge to meet Next Generation Science Standards for Natural Selection and Evolution (Box 1; (NGSS Lead States 2013)). Curriculum materials are available as Additional files 2, 3 and 4.

Box 1. Next Generation Science Standards (NGSS) Targeted by EvolvingSTEM NGSS (2013) are based on A Framework for K-12 Science Education: Practices, Crosscutting Concepts, and Core Ideas (National Research Council 2012) and designed through a collaboration between 26 states, the National Research Council, the National Science Teachers Association, the American Association for the Advancement of Science, and Achieve, Inc.

EvolvingSTEM provides students with the knowledge to meet the following NGSS HS-LS4 standards. These are performance expectations.

1. Communicate scientific information that common ancestry and biological evolution are supported by multiple lines of empirical evidence.
2. Construct an explanation based on evidence that the process of evolution primarily results from four factors: (1) the potential for a species to increase in number, (2) the heritable genetic variation of individuals in a species due to mutation and sexual reproduction, (3) competition for limited resources, and (4) the proliferation of those organisms that are better able to survive and reproduce in the environment.
3. Apply concepts of statistics and probability to support explanations that organisms with an advantageous heritable trait tend to increase in proportion to organisms lacking this trait.
4. Construct an explanation based on evidence for how natural selection leads to adaptation of populations.
5. Evaluate the evidence supporting claims that changes in environmental conditions may result in (1) increases in the number of individuals of some species, (2) the emergence of new species over time, and (3) the extinction of other species.

In addition, for HS-LS4-2, students will learn:

- Random mutation results in genetic variation between members of a population.
- Genetic variation can result in trait variation that leads to performance differences among individuals.
- Competition for limited resources results in differential survival. Individuals with more favorable phenotypes are more likely to survive and reproduce, thus passing traits to subsequent generations.
- Evolutionary fitness is measured by reproductive success.
- An adaptation is a heritable genetic variant manifested as a trait that provides an advantage to an individual in a particular environment.
- In addition to natural selection, chance and random events can influence the evolutionary process, especially for small populations.

In addition, students will be skilled at:

- Developing experimental investigations that can be used to test specific hypotheses.
- Evaluating evidence to qualitatively and quantitatively investigate the role of natural selection in evolution.
- Constructing evidence-based explanations that the process of evolution is a consequence of the interaction of four factors: (1) the potential for population size to increase, (2) genetic variation, (3) competition for resources, and (4) proliferation of individuals better able to survive and reproduce in a particular environment.
- Applying basic mathematics to calculate the fitness advantages of selected mutants and/or to compare differences in levels of biofilm production.
- Developing generalizations of the results obtained and/or the experimental design and applying them to new problems, including the design of new experiments and interpreting results in the context of natural and infectious bacterial biofilms.

### Learning outcomes

The exact outcome of any individual experiment is unknown because the biofilm selection acts on randomly occurring mutations in the bacterial populations that were founded from a single clone. In fact, this variability among these independent “replays” of evolution is realistic and demonstrates effects of chance and contingency on evolution (Blount et al. 2018). Nonetheless, student groups propagate multiple populations in different culture tubes under identical experimental conditions, and this replication means they are very likely to see mutants with novel morphologies in at least one experimental population. In addition, students compare their experimental populations to a control population that does not contain the bead and therefore is not under selection for increased biofilm production. Students can examine the phenotypes found in each population over time, compare their findings to those of other classmates, and develop their own explanations for their observations. This allows students to apply the comparative method of evolutionary biology and begin the process of scientific inquiry. Students are encouraged to consider why their replicate populations vary and propose reasons for that variation, ranging from experimental error, to peculiarities of the bead transfers, to genuine evolutionary randomness.

The speed of adaptation in biofilm models results from strong selection for more adherent mutants that bind not only the provided surface (e.g. polystyrene), but also other attached bacteria or secreted substances. Consequently, selection often favors the evolution of diverse, conspicuous phenotypes within each tube and not just a single, more adherent type. This result not only simulates the process of adaptive radiation often illustrated using Darwin’s finches in textbooks (Fig. 2), but also reproduces the selection for traits associated with adherence that often occurs during biofilm-associated infections (Traverse et al. 2013; Cooper et al. 2014; O’Rourke et al. 2015; Gloag et al. 2018). The “wrinkly” colony morphologies that evolve in our model are genetically and functionally identical to those commonly isolated from infections of the related species *Pseudomonas aeruginosa* in the airways of cystic fibrosis patients and in chronic skin wounds (Starkey et al. 2009; Gloag et al. 2018). Students can therefore connect their classroom experiments to recent findings at the interface of evolutionary biology and medicine to see how basic biological research impacts their everyday lives. Furthermore, making connections from classroom activities to real-world examples can increase students’ understanding of evolution and their engagement with the material (Beardsley et al. 2011; Infanti and Wiles 2014).

### Assessment of student learning

We used a delayed intervention approach to assess learning in 4 classes of 9th grade biology honors students at Winnacunnet High School, a suburban public high school in New England. Group 1 included classroom A, taught by MH, and classroom B, taught by SS. This group used an earlier version of our EvolvingSTEM curriculum that did not use a control population alongside their standard curriculum materials, which included textbook readings, lectures, and an educational video. Group 2 included classrooms C and D, both taught by SS. This group first received the standard curriculum with additional lecture materials, followed by EvolvingSTEM (Table 1). Students conducted the experiments and analyses for our curriculum in groups of three or four individuals, requiring collaborative teamwork.

**Table 1:** Composition of study groups

Group	Class—teacher	Number of students per class	Total number of students per group
1	A—Teacher MH	19	41
	B—Teacher SS	22	
2	C—Teacher SS	18	37
	D—Teacher SS	19	

A summative assessment was used to determine whether students achieved an increased understanding of evolutionary concepts. The test consisted of multiple choice and free response questions to address student learning of higher-order critical thinking aligned to NGSS. Specifically, test questions were devised to assess whether students met NGSS (NGSS Lead States 2013) performance expectations HS-LS4-1, 2, 3, and 5. We developed a grading rubric for the free response questions based on templates suggested by Wiggins and McTighe (2005) that required answers with accurate information, specific vocabulary, and a well-structured defense that incorporated outside examples (Wiggins and McTighe 2005). Our assessment and grading rubric are available as supplemental files (Additional file 5). All assessments were conducted by one of us (TW) on anonymized tests as proscribed by our IRB.

**Fig. 3:**
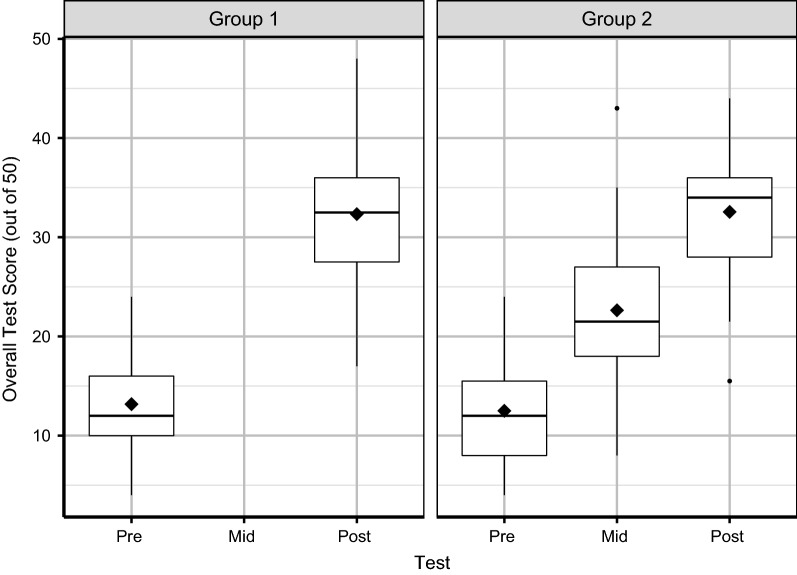
Boxplot of student assessment scores. The EvolvingSTEM curriculum produces significantly greater gains in comprehension of NGSS topic HS-LS-4 than the standard curriculum (Group 1 Post vs Group 2 Mid, t = 5.87, p < 0.0001). After experiencing our curriculum, Group 2 students subsequently achieved equivalent scores to Group 1 students (Group 1 Post vs Group 2 Post, t = 0.14, ns). Mean values are indicated with diamonds

Pretests were given to both groups prior to the start of classroom evolution activities. Group 1 students were given a posttest after completing the EvolvingSTEM curriculum. Group 2 students were given a midtest after completing the standard curriculum, and then a posttest after completing EvolvingSTEM. We found no significant difference between the average pretest score of Group 1 and Group 2 students (13.17 (26%) vs. 12.5 (25%) out of 50 points total; t = 0.60, p = n.s.), indicating that all students began with a similar knowledge base (Fig. 3). Quantitative analyses of student knowledge gains revealed that students who completed EvolvingSTEM (Group 1) showed significant improvement on their average posttest scores, with an average gain of 19.16 points, thereby increasing their overall score by 38% between the pre- and posttest (t = 16.61, p < 0.0001). Students provided the standard curriculum (Group 2) also showed significant improvement on their average midtest score, which increased by 10.14 points (t = 9.72, p < 0.0001), resulting in an overall increase of 21% between pre- and midtest. Although both student groups showed improvement, Group 1 achieved significantly higher average test scores after completing EvolvingSTEM than Group 2 did after completing the standard curriculum (t = 5.87, p < 0.0001). Students who learned evolution with EvolvingSTEM therefore achieved significantly greater gains in comprehension of evolution than students who learned it from the standard curriculum.

Once students in Group 2 were exposed to EvolvingSTEM, their average posttest scores increased by 20% in comparison to their midtest scores, reaching knowledge gains made by Group 1 students (Fig. 3). Knowledge gains by both Groups were overwhelmingly attributable to increased scores on the free-response section of the assessment. Average free-response scores from pretests to posttests increased by 18.09 points (48%) for Group 1 students and 20.59 points (54%) for Group 2 students. In comparison, average multiple-choice scores increased by 1.07 points for Group 1 students and decreased by 0.54 points for Group 2 students. These results may indicate that EvolvingSTEM has a greater impact on improving students’ higher-order cognitive skills, such as applying knowledge to an unknown problem and performing data analysis. There was no significant difference between Group 1 and 2 posttest scores (t = 0.14, p = n.s.), even though Group 2 students were provided more detailed verbal instruction and took one additional assessment. This result speaks to the power of EvolvingSTEM to increase student knowledge and suggests that our curriculum can serve to replace, rather than supplement, the standard evolution curriculum.

## Discussion

We developed an inquiry-based microbiology curriculum to improve the engagement of high school biology students with topics central to evolutionary biology and their subsequent understanding of related NGSS concepts. We observed high levels of engagement when students participated in our curriculum. Students were assigned concept and readiness tests each night to ensure that they arrived prepared for the next day’s microbiology experiments and evolution curriculum (Additional file 4). Their high rates of completion indicated increased enthusiasm. While we acknowledge this is a simple observation, teachers and coauthors (MH and SS) also indicated that students who rarely participated in class-based discussions emerged as enthusiastic group leaders while performing the EvolvingSTEM experiment. Informal post-surveys of student attitudes towards the curriculum were overwhelmingly positive. Students indicated that they were enthusiastic about the bacterial model, enjoyed coming to class to work on the experiment, and felt that our curriculum was better at teaching them than the standard lecture-style class. The group format for the experiments and analyses encouraged the students to collaborate and support one another throughout the program. Students tended to hold one another accountable, but also demonstrated cohesion when groups compared their replicate populations, demonstrating both friendly competition and pride and ownership in their results. Further, many students expressed that they felt like “real scientists” using equipment like pipettes, vortexes, and the incubator. They shared a greater sense of what science was actually like and asked more questions about microbiology and evolution research and other scientific careers.

Crucially, teachers found EvolvingSTEM to be effective at demonstrating evolution in action, thereby increasing student understanding of natural selection, mutation, and the effects of chance, and increasing student interest and engagement with biology. Student assessments also demonstrated the substantial benefit of our curriculum to student learning, and consequently, our curriculum replaced the standard, honors biology WHS evolution curriculum in subsequent years. The sustainability of the EvolvingSTEM curriculum has been greatly facilitated by the involvement of returning students who demonstrated particular interest in the program and who served as de facto teaching assistants through an Extended Learning Opportunity program. (More information about this program will be the subject of a future report.) This teaching experience was made possible by engaging first-year students in laboratory research, which allowed them to help teach new students for up to three subsequent years prior to graduating.

We found that EvolvingSTEM provided students with significant learning benefits in comparison to standard curricula. After completing our curriculum, students achieved significantly higher scores on a knowledge assessment of evolution than students who had followed the standard curriculum. After completing our curriculum, students who were originally provided only the standard curriculum were able to further increase their assessment scores to meet the gains made by students who were taught evolution only with EvolvingSTEM. Our results demonstrate the power of microbial evolution experiments to effectively teach concepts in population genetics and evolution while also providing valuable experience in microbiology. Furthermore, EvolvingSTEM can serve as an instructional foundation of other life science topics. For example, further investigations by students could identify the genetic mutations (using inexpensive whole-genome sequencing, i.e. (Cooper 2018)) that underlie the adaptive mutant phenotypes, supporting a greater understanding of inheritance and trait variation (NGSS HS-LS3). Previous research in our lab indicates that many commonly identified mutations are found in the *wsp* (wrinkly spreader phenotype) gene cluster (Cooper et al. 2014; Gloag et al. 2018), which coordinates bacterial surface recognition with increased biofilm production (Hickman and Tifrea 2005). Students are likely to identify *wsp* mutants in their classroom experiments and can therefore connect how changes in DNA can result in changes in protein structure and intracellular signaling that lead to increased biofilm production and changes to colony morphology, supporting a greater understanding of DNA, protein structure, and cellular function (NGSS HS-LS1). Furthermore, the bacterial adaptations are in response to environmental changes that provide new niches, supporting a greater understanding of interdependent relationships in ecosystems (NGSS HS-LS2). Classroom experiments that build upon the core evolution study can therefore span much of the NGSS-recommended introductory biology curriculum and have been adapted to cover more advanced topics for Advanced Placement (AP) Biology as well as to early biology courses in community colleges or 4-year colleges.

This study was limited to one school and two teachers from a suburban public school in New Hampshire, which naturally raises the question of its efficacy in other settings. However, since the program launch and assessments reported here, EvolvingSTEM has expanded to be offered in 13 high schools in four different US states with continued growth. These schools range from independent private schools, to suburban public schools, to urban public and magnet high schools, and the classes include introductory “academic” and honors biology, upper-level biotechnology, and AP biology. The core experimental protocol described here has been shown to be robust to different class schedules and student populations, provided that the classroom has the laboratory resources detailed in Additional file 1, including the capacity to prepare sterile growth media either onsite or through a partner laboratory. Additional assessments of learning and motivation towards STEM subjects are ongoing in these schools, but informal teacher and student feedback has been overwhelmingly positive.

## Summary

EvolvingSTEM is an engaging, inquiry-based curriculum that provides students with a hands-on approach to visualize evolutionary change occurring in real time. It also can be delivered at a low cost per student (< $5 in consumables) and is therefore potentially suitable for broad distribution. Our curriculum provides students with the tools to understand evolutionary concepts and to apply their knowledge to other areas of life science and medicine. For example, students can make a direct link between the adaptive phenotypes they see in the classroom for increased biofilm production and the nearly identical phenotypes seen in clinically relevant biofilm-associated bacterial infections. In addition, students are provided an introduction to microbiological techniques that have important applications for biotechnology. A particularly powerful aspect of our curriculum is its positive effect on teacher and student engagement. Teachers and students embark on the research experiment together, which provides a collaborative classroom environment where both have the opportunity for greater understanding and discovery. EvolvingSTEM has exceptional ability to improve scientific literacy and the promise of promoting broad acceptance of evolution as a central, unifying theory for life science.

## Additional files

**Additional file 1.** Materials needed and media recipes.

**Additional file 2.** EvolvingSTEM experimental protocol.

**Additional file 3.** Curriculum overview.

**Additional file 4.** Pre and post lab questions.

**Additional file 5.** Student test and grading rubric.
