# Supplementary material for: EvolvingSTEM: a microbial evolution-in-action curriculum that enhances learning of evolutionary biology and biotechnology
Source: Evolution (N Y). 2019 Apr 24;12(1):12. doi: 10.1186/s12052-019-0103-4 (PMC7328067; doi:10.1186/s12052-019-0103-4)
Supplement: Supplementary file 1 — Additional file 1. Materials needed and media recipes. [file 12052_2019_103_MOESM1_ESM.pdf]

Supplemental Files

Sup. 1: Materials Needed and Media Recipes

Sup. 2: EvolvingSTEM Experimental Protocol

Sup. 3: Curriculum Overview

Sup. 4: Pre and Post Lab Questions

Sup. 5: Student Test and Grading Rubric

S1. Materials Needed per Classroom

Materials for entire classroom

- Gloves (will need at least 6 pairs per student)
- Spray bottle of 70% Ethanol (to clean benchtop)
- Spray bottle of 10% Bleach (to decontaminate bacterial cultures and plates)
- Orbital shaker
- Incubator
- Serological pipettes and Pipette aid (to prefill tubes with media and PBS)
- *Pseudomonas fluorescens* SBW25 colonies streaked on ½ Tsoy-Agar plates (need to have 4 distinct colonies for each student group)
- Autoclave (to sterilize reusable materials and media)
- Dissecting microscope (not required, but helpful to visualize colonies)

Materials for each student group

- Bunsen burner
- Vortex
- Micropipettes and tips: p200 and p1000
- Forceps: 1 pair
- Metal inoculation loop: 1
- Glass spreader beads
- Glass culture tubes (15mL): 36
- Small glass tubes (5mL): 8
- 5 and 15 mL tube racks
- White beads: 9
- Black beads: 3
- Queen's B Media: 82mL
- PBS: 102mL
- Tsoy-agar plates: 12

Media Recipes

1L Queen's B Media
- 20g Proteose Peptone No. 3
- 1.5g K_2HPO_4 (Potassium Phosphate Dibasic)
- 25mL Glycerol
- 970mL Water

1. Autoclave for 45 minutes
2. Allow to cool to room temperature
3. Add 6mL of 1M $MgSO_4$ (Magnesium Sulfate) stock

250mL 1M $MgSO_4$ Stock
- 30g $MgSO_4$ (anhydrous)

  or
- 61.6g $MgSO_4$ (heptahydrate)
- 250mL Water

1. Combine salts and water
2. Autoclave for 30-45 minutes

1L PBS
- 7.65g NaCl
- 0.72g Na_2HPO_4 (Sodium Phosphate Dibasic, anhydrous)
- 0.21g KH_2PO_4 (Potassium Phosphate Monobasic)
- 1L Water

1. Combine salts and water
2. Autoclave for 45 minutes

1L ½ Strength Tsoy-Agar (makes approximately 50 Plates)
- 15g Tsoy
- 15g Agar
- 1L Water

1. Autoclave for 45 minutes
2. Pour plates while still hot (so agar does not harden)
3. Allow to solidify overnight before using
