# Supplementary material for: EvolvingSTEM: a microbial evolution-in-action curriculum that enhances learning of evolutionary biology and biotechnology
Source: Evolution (N Y). 2019 Apr 24;12(1):12. doi: 10.1186/s12052-019-0103-4 (PMC7328067; doi:10.1186/s12052-019-0103-4)

File S2.

EvolvingSTEM

Pseudomonas fluorescens Experimental Evolution Protocol

You are about to embark on a journey through a world that you might be unfamiliar with; one filled with odd instruments that you will use to study oddly shaped slimy bacterial colonies and neon yellow biofilm-coated test tubes. Over the course of the next few weeks you will be taking care of bacterial cultures, and your ordinary looking colonies will evolve to produce distinct mutants that have adapted to inhabit different parts of a test tube.

ALWAYS REMEMBER

Proper **aseptic technique** is a very important part of microbiology! All tubes, beads, and media have been sterilized in an **autoclave** prior to use in these experiments. When tubes were prepared, media was always distributed using sterile pipettes, and sterile beads were added using forceps that have been heated over a flame until *"red-hot"* to prevent contamination.

USEFUL TERMS

Aseptic Technique – a sterile set of practices and procedures performed to minimize contamination by other bacteria.

Autoclave – a strong, heated container that reaches high temperature and pressure to sterilize equipment and media.

SAFETY FIRST!

You will be working with an open flame during this experiment. Always be aware of your surroundings to ensure that you do not burn yourself or start a fire. Be sure to know the location of the closest emergency shower and fire extinguisher in case an accident does occur.

Always treat the bacteria you will be working with as potential pathogens (even though *P. fluorescens* is harmless to humans!). Be sure to disinfect your work stations and waste materials with 10% bleach and always follow general safe lab procedures, including tying back long hair, washing your hands at the end of the lab activity, and wearing gloves and lab coats.

The following links provide excellent information on:
General Lab Safety: https://www.youtube.com/watch?v=MEIXRLcC6RA&vl=en
Safely Working with Microorganisms: https://www.sciencebuddies.org/science-fair-projects/references/microorganisms-safety

DAY 1 (**MONDAY): PRECONDITIONING YOUR BACTERIAL CULTURE**

Before the bacteria got to your classroom, they had been stored in a freezer for a long period of time at -80° Celsius (-112° Fahrenheit). Before we can continue with our experiment, we want to ensure that the bacteria are used to being out of the freezer so they are performing at their prime. In order to do so, we give them time to get acclimated to their new environmental conditions – this day is known as our "Preconditioning Day".

NECESSARY MATERIALS

- Inoculation loops
- *Pseudomonas fluorescens* SBW25 colonies (on agar plate)
- 3 Large glass culture tubes containing:
  - 5 mL Queen's B Medium (QB)
  - 1 white polystyrene bead
- 1 Large glass culture tube containing:
  - 5 mL Queen's B Medium (QB)

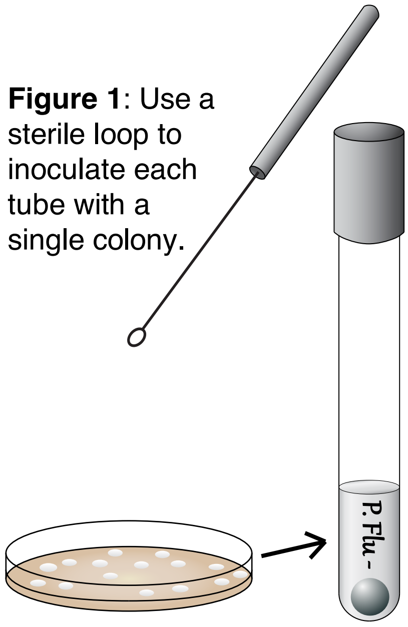

Figure 1: Use a sterile loop to inoculate each tube with a single colony.

PROCEDURE:

1. Use an inoculation loop to transfer a **single** isolated *P. fluorescens* colony to a **single** culture tube.

2. Repeat until you have inoculated all four tubes: "1", "2", "3", "C".
   BE SURE TO USE A NEW COLONY TO INOCULATE EACH TUBE!

3. Incubate the culture tubes on a rotating shaker at 28°C until your next class.

DAY 2 (TUESDAY): BEAD TRANSFER AND PLATING

NECESSARY MATERIALS:

- Metal forceps
- 4 Small glass tubes containing 1 mL QB
- Vortex
- A p200 and p1000 pipette
- 3 Large glass evolution tubes containing:
  4.5 mL QB
  1 **white** polystyrene bead
- 1 Large glass control tube containing:
  4.5 mL QB
- 8 Large glass culture tubes containing 5 mL Phosphate Buffered Saline (PBS)
- 4 ½ Strength Tsoy-Agar plates with small glass beads

PROCEDURE:

1. Label the large glass culture tubes and agar plates in an identifiable manner.

2. Flame sterilize the forceps and allow them cool for 30 seconds.

   After flaming the forceps they must not touch anything else, or they are no longer considered to be sterile!

3. **For each evolution culture:** Pour the contents of the culture tube into its metal cap, and then use sterile forceps to transfer ONLY the bead to the corresponding small QB tube.

   It is possible that you may hear a sizzle; this is normal and just means that the forceps are still hot from sterilization. Allow them to cool until you no longer hear a sizzle before you touch the polystyrene bead.

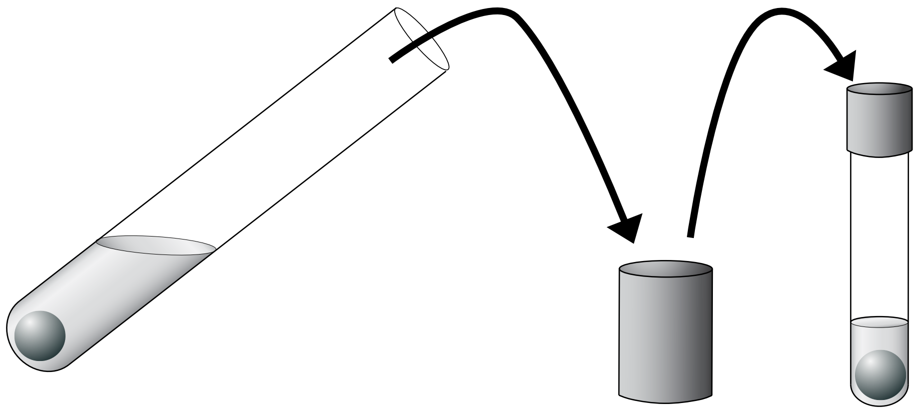

Figure 2: The preconditioning culture (left) is poured into its cap, and then sterile forceps are used to move only the bead to the small QB tube (right).

Vortex the small QB tube for at least 45 seconds to remove biofilm from the bead.

For the control tube: Briefly swirl the control culture tube, and then use a **p200** pipette to transfer 50 μl of the culture to the small QB tube. Briefly vortex the small QB tube.

Perform the following steps for all evolution and control cultures:

4. Use a **p1000** pipette to transfer 500 μl from the small QB tube to the large QB tube. Briefly vortex to mix.

5. Use a **p200** pipette to transfer 50 μl from the large QB tube to a PBS tube (10^{-2} dilution). Briefly vortex to mix.

6. Use a **p200** pipette to transfer 50 μl from the 10^{-2} tube to a new PBS tube (10^{-4} dilution). Briefly vortex to mix.

7. Use a **p200** pipette to transfer 100 μl of the 10^{-4} dilution to an agar plate.

8. Shake the plate with the lid on top using the glass beads to spread the liquid culture. Remove the glass beads by turning the plate upside down and dumping the beads from the lid into the container provided.

9. Incubate the culture tubes on a rotating shaker at 28°C until your next class. Incubate the plates, **upside down**, at 28°C until your next class.

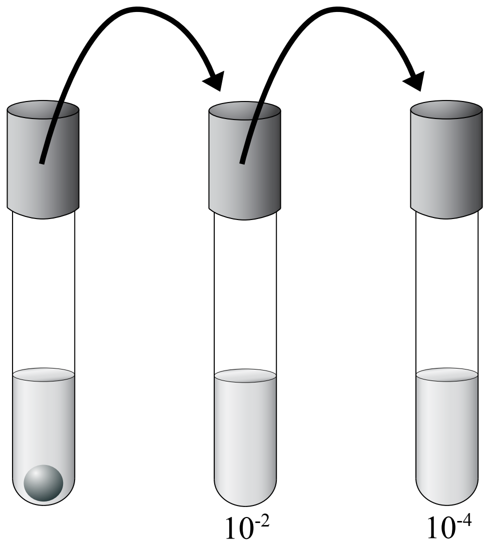

Figure 3: Serial dilution from the evolution tube (left) into PBS.

DAY 3 (WEDNESDAY): BEAD TRANSFER

The millions of cells that you added to your tube will quickly grow to become billions. It doesn't take long before the bacteria consume the food and nutrients provided by the media inside of the test tube. In order to make sure that the bacteria continue to survive, we have to transfer a small number into a new tube. In the case of our experimental cultures, we transfer only the bacteria that are good at forming biofilm and have thus successfully stuck to the bead.

NECESSARY MATERIALS:

- Metal forceps
- Vortex
- p200 pipette
- 3 Large glass evolution tubes containing:
  5ml QB
  1 **black** polystyrene bead
- 1 Large glass control tube containing:
  5ml QB

PROCEDURE:

1. Flame sterilize and cool the forceps.

2. **For each evolution culture:** Pour the contents of the culture tube into its metal cap, and then use sterile forceps to transfer the **white bead** to a new evolution tube with fresh media and a **black bead**.

   For the control culture: Briefly swirl the control culture tube, and then use the **p200** pipette to transfer 50 μl of the culture to the new control tube.

3. Incubate the culture tubes on a rotating shaker at 28°C until your next class.

DAY 4 (THURSDAY): BEAD TRANSFER

You may have noticed that your incubated test tubes now contain both a white and a black bead. Today, you are transferring your black bead to a new tube containing fresh media and a white bead. Over time, some of the bacteria from the black bead will detach and re-adhere to the surface of the white bead.

NECESSARY MATERIALS:

- Metal forceps
- Vortex
- p200 pipette
- 3 Large glass evolution tubes containing:
  5ml QB
  1 **white** polystyrene bead
- 1 Large glass control tube containing:
  5ml QB

PROCEDURE:

1. Flame sterilize and cool the forceps.

2. **For the evolution tubes:** Pour the contents of the culture tube into its metal cap, and then use sterile forceps to transfer the **black bead** to the new corresponding evolution tube with fresh media and a **white bead**.

   For the control tube: Briefly swirl the control tube culture, and then use the **p200** pipette to transfer 50 μl of the culture to the new control tube.

3. Incubate the culture tubes on a rotating shaker at 28°C until your next class.

DAY 5 (FRIDAY): FINAL PLATING

NECESSARY MATERIALS:

- Metal forceps
- Vortex
- A p200 and p1000 pipette
- 4 Small glass tubes containing 1 mL Phosphate Buffered Saline (PBS)
- 8 Large glass culture tubes containing 5 mL Phosphate Buffered Saline (PBS)
- 4 Large glass culture tubes containing 4.5 mL Phosphate Buffered Saline (PBS)
- 8 ½ Strength Tsoy-Agar plates with glass beads

PROCEDURE:

1. Flame sterilize and cool the forceps.

2. **For each evolution tube:** Pour the contents of the culture tube into its metal cap, and then use sterile forceps to transfer the **black bead** to the small glass tube with PBS. Vortex the small PBS tubes for at least 45 seconds to remove cells from the bead.

   For the control tube: Briefly swirl the control tube culture, and then use a **p200** pipette to transfer 50 μl of the culture to a small glass tube with PBS. Briefly vortex the small PBS tube.

Perform the following steps for all evolution and control cultures:

3. Use a **p200** pipette to transfer 50 μl from the small PBS tube to a 5mL PBS tube (10^{-2} dilution). Briefly vortex to mix.

4. Use a **p200** pipette to transfer 50 μl from the 10^{-2} tube to a new 5mL PBS tube (10^{-4} dilution). Briefly vortex to mix.

5. Use a **p1000** pipette to transfer 500 μl of the 10^{-4} tube to the 4.5mL PBS tube (10^{-5} dilution). Briefly vortex to mix.

6. Use a **p200** pipette to transfer 100 μl of the 10^{-4} and 10^{-5} dilution tubes to agar plates

7. Shake the plates with their lids on top using the glass beads to spread the liquid culture. Remove the glass beads by turning the plates upside down and dumping the beads from the lid into the container provided.

8. Incubate the plates, **upside down**, at 28°C until your next class.

DAY 6 (MONDAY): COLONY EXAMINATION

NECESSARY MATERIALS:

- Dissecting microscope

PROCEDURE:

Closely examine colony morphology:

- Do all colonies look exactly the same as those plated last Monday?
- If not, how many are different?
- Describe the following for each colony type:
  - Size – large, medium, or small
  - Texture – smooth or rough
  - Color
  - Shape

Use the following chart to help describe changes in colony appearance:

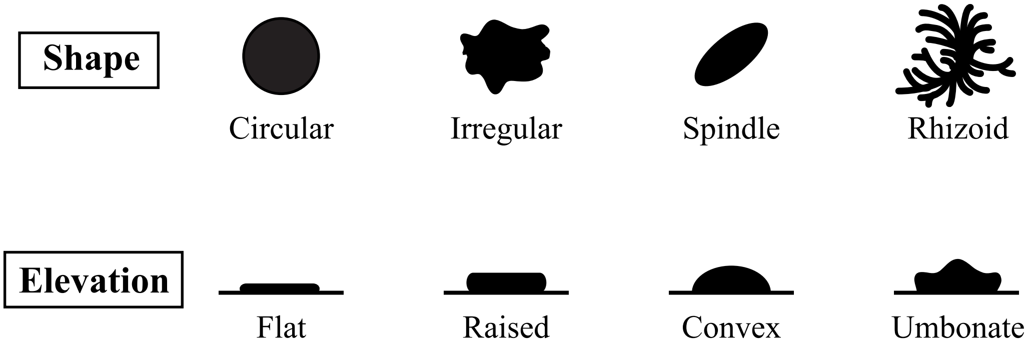

Supplement: Supplementary file 2 — Additional file 2. EvolvingSTEM experimental protocol. [file 12052_2019_103_MOESM2_ESM.pdf]
