# Supplementary material for: EvolvingSTEM: a microbial evolution-in-action curriculum that enhances learning of evolutionary biology and biotechnology
Source: Evolution (N Y). 2019 Apr 24;12(1):12. doi: 10.1186/s12052-019-0103-4 (PMC7328067; doi:10.1186/s12052-019-0103-4)
Supplement: Supplementary file 3 — Additional file 3. Curriculum overview. [file 12052_2019_103_MOESM3_ESM.pdf]

File S3. EvolvingSTEM Curriculum Overview

<table>
<tr><th colspan="3">Stage 1 Desired Results</th></tr>
<tr><td rowspan="4">ESTABLISHED GOALS

The process of evolution drives the diversity and unity of life.

• Big Idea 1 – AP Bio
• HS-LS4 – NGSS</td><td colspan="2">Transfer</td></tr>
<tr><td colspan="2">Students will be able to independently use their learning to complete a performance task at a Depth of Knowledge Level 3 and/or 4 (DOK 3 & DOK 4)

Depth of Knowledge Level 3 (Strategic Thinking)
• Justify a response when more than one answer is possible
• Cite evidence and develop a logical argument for concepts
• Design and conduct an investigation
• Research and explain a scientific concept

Depth of Knowledge Level 4 (Extended Thinking)
• Based on provided data from a complex experiment that is novel to the student, deduct the fundamental relationship between several controlled variables
• Conduct an investigation, from specifying a problem to designing and carrying out an experiment, to analyzing its data and forming conclusions
• Develop generalizations of the results obtained and the strategies used and apply them to new problem situations</td></tr>
<tr><td colspan="2">Meaning</td></tr>
<tr><td>UNDERSTANDINGS
Students will understand that…
• Change in the genetic makeup of a population over time is evolution.
• Organisms are linked by lines of descent from common ancestry.
• Life continues to evolve within a changing environment.</td><td>ESSENTIAL QUESTIONS
1. What is evolution?
2. Do humans influence evolution?
3. What is natural selection?
4. How does natural selection lead to adaptation of populations?
5. How can microbiology be used to understand the mechanisms of</td></tr>
</table>

<table>
<tr><td rowspan="3"></td><td>(Enduring Understandings 1.A, 1.B, 1.C – AP Biology)</td><td>adaptation and evolution?
6. What is the difference between adaptation and evolution?</td></tr>
<tr><td colspan="2">Acquisition</td></tr>
<tr><td>Students will know…
• Competition for limited resources results in differential survival. Individual with more favorable phenotypes are more likely to survive and reproduce more offspring, thus passing traits to subsequent generations.
• Evolutionary fitness is measured by reproductive success.
• Genetic variation and mutation play roles in natural selection.
• An adaptation is a genetic variation that is favored by selection and is manifested as a trait that provides an advantage to an organism in a particular environment.
• In addition to natural selection, chance and random events can influence the evolutionary process, especially</td><td>Students will be skilled at…
• Developing experimental designs that can be used to test specific hypotheses.
• Evaluating evidence provided by data to qualitatively and quantitatively investigate the role of natural selection in evolution.
• Constructing evidence-based explanations that the process of evolution results from four primary factors.
• Applying basic math calculations related to experiments.

Science and Engineering Practices
• Analyzing and interpreting data
• Using mathematics and computational thinking
• Constructing explanations and designing solutions
• Engaging in argument from evidence</td></tr>
</table>

	for small populations.<br>• Humans impact variation in other species.<br>• Biochemical and genetic similarities, in particular DNA nucleotide and protein sequences, provide evidence for evolution and ancestry.<br>• DNA and RNA are carriers of genetic information through transcription, translation, and replication.<br><br>Connections to Nature of Science<br>• Scientific knowledge assumes an order and consistency in natural systems.<br>• Science models, laws, mechanisms, and theories explain natural phenomena.	• Obtaining, evaluating, and communicating information<br><br>Crosscutting Concepts<br>• Observing different patterns at each of the scales at which a system is studied.<br>• Utilizing empirical evidence to make claims about specific causes and effects.

**Stage 2 – Evidence**	
**Evaluative Criteria**	**Assessment Evidence**
Project Rubric<br>Peer evaluation<br>Self-evaluation<br>Presentation with rubric<br>• science showcase<br>• school board meeting<br>• conference<br>• step up day<br>• vertical team meeting	PERFORMANCE TASK(S):<br><br>1. Given specific experimental conditions, predict the outcome of bacterial growth supported by existing research. (2-3 days).<br><br>2. Student-designed projects to change experimental conditions such as:

• middle schools/other high schools<br>• in class	• Intermediate levels of disturbance – shaking, non-shaking, mix between shaking and non-shaking.<br>• Altering nutrient levels<br>• Oxygen availability<br>• Balance between oxygen and nutrients – short and fat or long and thin microcosms<br>• Changing incubation conditions (2 weeks)
Journal grade with rubric<br>Daily participation grade (5 point/day)<br>Numerical test and quiz grades<br>Teacher and peer feedback<br>Self-reflection	OTHER EVIDENCE:<br>1. Lab Journal – daily entries<br>2. Class Starter and Exit Ticket questions – review previous day, lab protocol or technique, address misconceptions, standardized test release questions<br>3. Lab participation – daily grade<br>4. Probes as formative assessment to address misconceptions, identify areas of reteaching, and document student growth.<br>5. Quizzes and tests - document learning of content knowledge and lab skills

Stage 3 – Learning Plan

Summary of Key Learning Events and Instruction

Learning Activities
A brief summary of the key learning activities is provided below.

Prior to starting EvolvingSTEM (optional)
- Meet *Pseudomonas fluorescens* – read a bio on *P. flourescens*, brainstorm as a group the similarities and differences between *P. flu* and humans; introduce that common ancestry and biological evolution are supported by multiple lines of empirical evidence
- Complete geologic timeline to introduce deep time
- Introduce lab techniques – swab the world, pipetting competitions, Bunsen burner lighting and safety (Videos available – online or DVD)
- Journal articles – biofilm, CF connection, *P. flourescens* background

During EvolvingSTEM Experiment
- Refer to daily calendar for planning/scope and sequence.

- Provide each student with a color-coded Evolution in Action protocol with daily procedures and questions for lab journal.
- Post and discuss essential questions and understanding.
- Use lab and content-based videos (online or DVD) with questions to maximize class time in the lab. Videos can be used in a flipped classroom model, in class model, or a blend of both.
- Introduce class starter and exit ticket questions/probes to assess student learning, address misconceptions, and review material.
- Review probes and lead discussions as necessary to address and correct misconceptions.
- Schedule "Ask The Expert" with Dr. Cooper (Skype) to introduce research, CF connection, field student questions.
- Use standard assessments (quiz/test) to gauge student learning as they progress through the unit. (formative and summative)
- Assign scientific journals and readings on evolution (Common Core Literacy Standard). Lead small and/or large group discussions, fish bowl, whiteboard report out.
- Introduce Performance Task. Discuss rubric and timeline.
- Engage students in peer and self-evaluation.
- Organize student showcase to display and discuss work.

Optional Extensions

1. Students will review scientific literature that describes the mutations associated with given phenotypes. Genomic sequencing of these phenotypes will allow them to make connections between the literature and their findings (Common Core Literacy Standard).

2. Research project/Webquest – Explore similarities and differences of two or more genomes (NCBI, BLAST, NIH) to begin to understand common ancestry

3. Explore STEM-related fields of study and careers in such areas as microbiology, physiology, engineering, medicine, and public health

4. Cystic Fibrosis – www.cff.org, CF walk participation, school team - fundraising, research, Webquest, Cooper Lab visit

5. SEM images of ancestral and mutant colonies – micrometer measurements, observations, compare and contrast

6. Exploration and discussion of antibiotic resistance

7. Fitness assays and genomic sequencing

8. Connections to other units – Genetics, Classification, Nature of Science (Inquiry), Cells, Characteristics of Life
