# Supplementary material for: EvolvingSTEM: a microbial evolution-in-action curriculum that enhances learning of evolutionary biology and biotechnology
Source: Evolution (N Y). 2019 Apr 24;12(1):12. doi: 10.1186/s12052-019-0103-4 (PMC7328067; doi:10.1186/s12052-019-0103-4)
Supplement: Supplementary file 4 — Additional file 4. Pre and post lab questions. [file 12052_2019_103_MOESM4_ESM.pdf]

File S4: Pre and Post-Lab Question
PRE-LAB QUESTIONS: DAY 1

1. In one sentence briefly describe the purpose of the “pre-conditioning” step that will be carried out on Day 1 of your experiment? How many colonies are used to inoculate one test tube containing media and a white bead?

2. Suzie has just used a sterilized inoculating loop to obtain a single isolated colony of bacteria, which she then transferred into her test tube containing fresh media and a white bead. After she has put the cap of her newly inoculated test tube back on, she grabs the petri dish and goes to grab another colony. Before she can touch the inoculating loop to the petri dish, Larry stops her and tells her that she is doing it wrong. Which student is correct in this case, and why?

POST-LAB QUESTIONS: DAY 1

1. What is another name of an error that is introduced during the process of replication that results in a new DNA sequence? What is the end product of this newly formed DNA sequence in comparison to the original DNA sequence?

2. What are the two possible forces that can act on mutations that occur in DNA sequences? Describe in detail the difference between these two different forces.

3. Describe the characteristics of bacteria that make them advantageous when studying evolution?

4. Summarize the different stages that occur throughout the biofilm lifecycle. How does this relate to the bead transfer model that is used in the experiments?

PRE-LAB QUESTIONS: DAY 2

1. In one sentence briefly summarize the process of serial dilutions. What is happening to the overall population size of the bacteria as you carry out these dilutions and what is achieved by completing them?

2. Draw the series of steps that are required to complete a serial dilution on Day 2. Include the amount of liquid that is being transferred, the amount of liquid that is in the dilution tube, and the dilution that is achieved with each step. Circle the dilution(s) that will be plated on Day 2.

3. In one sentence briefly summarize the process of plating a bacterial culture. What is achieved by plating, and why is it incredibly important to ensure that you are plating on the agar side of the plate?

4. Once the bead has been transferred from the large glass evolution tube to the small glass tube containing 1 mL of Queen's B media, how long should the small glass tube be vortexed for? What is the purpose of vortexing?

5. In general, the large media tubes will contain 5 mL of media; however, on Day 2 the large glass evolution tube only contains 4.5 mL of media. Can you explain why this is the case?

POST-LAB QUESTIONS: DAY 2

1. Why is it important to transfer the bacteria every 24 hours? Draw a graph that illustrates the growth of a bacterial culture. Make sure to label your axes!

2. Provide a detailed hypothesis that describes what you think might occur in your test tube over the next 24 hours when your bacteria from inside the test tube are adhering to the new bead. Try to use the following vocabulary in your predictions: planktonic, biofilm, and overproduction and polystyrene bead.

PRE-LAB QUESTIONS: DAY 3

1. What is the color of the old bead that is being transferred from the 24-hour large glass evolution tube? What is the color of the new bead that is in the new large glass evolution tube?

2. Why is it important to disrupt the bead as little as possible during your daily bead transfer?

3. Describe what your test tube looked like on Day 2 when you put it in the incubator following your transfer. What do you think it will look like on Day 3 when you remove it from the incubator? Describe the amount of biofilm that is seen on the sides of the tube, the type of biofilm that is seen, and the color of the liquid media.

POST-LAB QUESTIONS: DAY 3

1. Describe in detail the three different types of mutations that can occur and the possible effect of that given type of mutation. Which two types of mutations are generally more common, and which is the least likely to occur?

2. What is being accomplished by transferring only the bacteria that have successfully attached to the bead? What is this type of selection called?

3. Provide a detailed hypothesis that describes what you believe might occur in your test tube over the next 24 hours when your bacteria are detaching from the old bead and adhering to the new bead. Use the following vocabulary in your predictions: overproduction, planktonic, biofilm, exponential, polystyrene bead, and mutation (both beneficial and neutral).

PRE-LAB QUESTIONS: DAY 4

1. What is the color of the old bead that is being transferred from the 24-hour large glass evolution tube?

2. Describe what your test tube looked like when you put it in the incubator following your transfer on Day 3. What do you think it will look like on Day 4 when you remove it from the incubator? Describe the amount of biofilm that is seen on the sides of the tube, the type of biofilm that is seen, and the color of the liquid media.

POST-LAB QUESTIONS: DAY 4

1. It is possible that when you removed your tubes today, only one of them has significantly more biofilm on the sides of the tubes and has a neon culture. As we discussed, this is a possible indication that you have a beneficial mutation in your population. Can you provide an explanation for why only one of your four replicates looks like this if you started with identical bacteria at the beginning of your experiment?

2. If we were to impose a greater force of artificial selection on the bacteria that we are studying, would it increase the number of mutations that we see in our experiment? Why or why not?

3. We already know that bacteria grow at an incredibly fast rate and can potentially overproduce, causing them to produce more bacteria inside the test tube than can survive. This over production leads to another phenomenon, which is another point of Darwin's Theory of Evolution by Natural Selection. Explain how this is occurring inside of the test tube and how it relates to overproduction.

4. Provide a detailed hypothesis that describes what you believe might occur in your test tube over the next 24 hours when your bacteria are detaching from the old bead and adhering to the new bead. Use the following vocabulary in your predictions: overproduction, planktonic, biofilm, exponential, competition, mutation (both beneficial and neutral), resources, space, polystyrene bead, nutrients, frequency, niche, and heritable genetic variation.

PRE-LAB QUESTIONS: DAY 5

1. What is the color of the bead that is going to be plated? Why isn't it necessary to transfer the other bead to a new evolution tube containing fresh media and an oppositely marked bead?

2. Draw the series of steps that are required to complete a serial dilution on Day 5. Include the amount of liquid that is being transferred, the amount of liquid that is in the dilution tube, and the dilution that is achieved with each step. Circle the dilution(s) that will be plated on Day 5.

3. Provide a detailed hypothesis as to why you believe it is necessary to dilute one step further on Day 5 than on Day 2.

4. Describe what your test tube looked like when you put it in the incubator following your transfer on Day 4. What do you think it will look like on Day 5 when you remove it from the incubator? Describe the amount of biofilm that is seen on the sides of the tube, the type of biofilm that is seen, and the color of the liquid media.

POST-LAB QUESTIONS: DAY 5

1. Did one of your tubes change drastically from Day 4? If you observed a change in one of your tubes, explain the differences that you are seeing. In addition, provide possible explanations as to why you have yet to see a change in your other replicates.

2. Discuss how mutations increase in frequency over time. You may draw a graph below to illustrate this process.

3. When you removed your tube from the incubator on Day 2, it had not appeared to change greatly from when you started your experiment. When you removed your tube from the incubator on Day 5, the culture was neon yellow. You are sure that when you plate today, you will definitely have mutants on your plate. Your group member also states that it is possible that you have mutants on your Day 2 plates. Is he/she correct? Discuss the potential results that may be observed.

PRE-LAB QUESTIONS: DAY 6

1. Predict what your Day 5 colonies will look like when you view them in the lab. How will they look different from the colonies you plated on Day 2?

2. As we discussed previously, it is possible that you may see multiple phenotypes on your agar plate during the course of your evolutions. Provide a hypothesis that might explain the role that each of these mutants is playing in the community.

POST-LAB QUESTIONS: DAY 6

1. Explain how two mutants with distinct phenotypes can inhabit the same test tube simultaneously. Be sure to incorporate the importance of an ecological niche in your answer.

1. Now that you have completed your evolution experiment, do you believe that evolution is fast or slow? Provide an explanation to support your answer.

2. You now have all four pieces that are required to support Darwin's Theory of Evolution by Natural Selection. Use all four to comprise an explanation that can support our example of microevolution that occurred in our test tube over the past week. Do you think that these same four points can be applied to a macro-evolutionary example?

3. Explain the difference between evolution and adaptation.
