# Supplementary material for: EvolvingSTEM: a microbial evolution-in-action curriculum that enhances learning of evolutionary biology and biotechnology
Source: Evolution (N Y). 2019 Apr 24;12(1):12. doi: 10.1186/s12052-019-0103-4 (PMC7328067; doi:10.1186/s12052-019-0103-4)
Supplement: Supplementary file 5 — Additional file 5. Student test and grading rubric. [file 12052_2019_103_MOESM5_ESM.pdf]

File S5. STUDENT TEST

Student ID Number: ___________________________
Block Number: _______________________________
Teacher: _____________________________________
Date: _______________________________________
(Understanding)
From the groups of characteristics below, identify the best answer for describing evolution. (2 pts. each)

1. Rate of evolution:
   a. Evolution does not happen, the rate of change in species is zero
   b. Fast – taking place in just a few generations
   c. Slow – taking thousands of generations or many thousands of years
   d. Evolution can be either Fast or Slow
2. The fundamental source of genetic variation among individual organisms is:
   a. Levels of nutrition that individuals receive
   b. Random mutations in DNA sequences or chromosomes
   c. Physical changes accumulated during an organism's lifetime
   d. Unexpected changes occurring during embryonic development
3. Amount of change in evolution:
   a. Evolution occurs rapidly, with quick appearance of new traits
   b. Evolution occurs at rates ranging from gradual to rapid
   c. Evolution occurs gradually by the accumulation of small changes over time
   d. Evolution does not happen so the amount of change is zero
4. Types of organisms that evolve:
   a. Evolution does not happen in any type of organism
   b. Evolution occurs in tiny organisms like bacteria and other single-celled species
   c. Evolution occurs in large organisms like palm trees, crabs, snakes, and giraffes
   d. Evolution occurs in all groups of organisms
5. Which example statement best describes evolutionary change?
   a. There are no observable changes in organisms over time
   b. A cat fed on a good diet grows larger than a cat fed on a poor diet
   c. A fair-skinned person tans during a summer
   d. Plants growing on a wet, lush island grow higher than plants on a dry, desert island
   e. The bill shape of birds changes because the hardness of the seeds they eat changes
6. For evolution to occur, which genetic characteristic must be present?
   a. There must be an even number of chromosomes
   b. Individual organisms in a population must appear different
   c. The differences in organisms must be capable to be passed to offspring

d. All of the characteristics listed above must be present

(Apply)

7. The New Mexico Whiptail (*Cnemidophorous neomexicanus)* is a parthenogenic lizard species from New Mexico and Arizona. The population consists entirely of females capable of laying viable eggs without fertilization. Many years ago, after receiving the necessary collecting permits, your teacher collected a single (1) New Mexico Whiptail and brought it to your school laboratory in NH. Your school laboratory is well-resourced with aquaria and mealworms, and everything needed to rear healthy whiptails. The first clutch yielded 10 offspring from the original whiptail and they were divided into two equal groups (A and B). Individuals in group A were marked by clipping the tip of the last digit on the left hind toe, and group B by the same procedure on the right hind toe. Note that the toe clipping has no effect on the ability of the lizard to survive in the environment, and was only used as a way to distinguish the two groups. Your teacher has been observing the mothers of the egg clutches, and as young whiptails hatch you clip the appropriate toe to assign them to the proper group of their mother. After 500 generations of living together with hatches and deaths occurring, the population size has grown to 340 whiptails with 272 in Group A, and 68 in Group B.

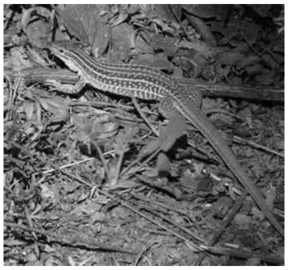

Count of Group A and B individual New Mexico Whiptails (*Cnemidophorous neomexicanus*) in the first generation (1) and in generation (500). Count does not include dead individuals, which were removed.

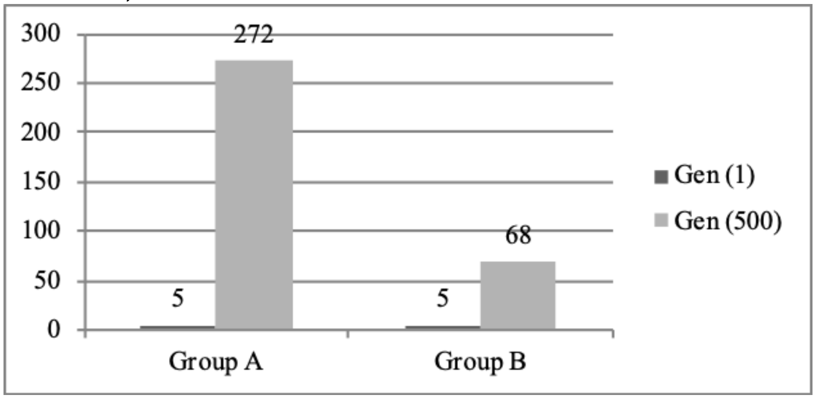

a. What is the percentage of Group A and Group B in the first generation of Whiptails? (2 points)

b. What is the percentage of Group A and Group B in the total population after 500 generations of hatches and deaths? (2 points)

c. Provide a possible evolutionary explanation for the shift in numbers of Group A and Group B individuals. (6 points)

____
____
____
____
____
____
____
____
____
____
____
____

8. List and briefly describe the 4 key elements that produce evolutionary change (12 points, 1.5 points for each correct concept, and 1.5 points for each correct description).

   1.

   2.

   3.

   4.

(Analyze)

9. You and your lab partner are given a test tube with a single living type of bacterium that grows in the water at room temperature. When you grow the organism in a petri dish, the bacterium only grows in circular- shaped colonies with clean, smooth edges. Then you grow your bacterium in the water but in a refrigerator. When you grow the organism from the refrigerator in a petri dish, you find circular-shaped colonies with clean, smooth edges, but also colonies with irregular-shapes and rough, jagged edges. You and your lab partner repeat this procedure, each time beginning with only the original bacterium. Each time you get the same result. Your lab partner exclaims, "The bacterium evolved!!" Is your lab partner correct? Why or why not? (16 points)

Pre- and Post-Test Grading Rubric

	EXCEEDS<br>4	MEETS<br>3	APPROACHES<br>2	BEGINS<br>1	STRUGGLES<br>0
Content Knowledge	Information on the topic is accurate. It addresses and extends beyond the questions raised in the prompt. Student incorporates outside examples that strongly help them defend their claims. Important vocabulary is used properly in the context of the response. It synthesizes competing ideas and says something new.	Information on the topic is accurate. It addresses the questions raised in the prompt. Important vocabulary is used, but not necessarily in the proper context.	Information on the topic is accurate, but does not address all questions raised in the prompt. Important vocabulary is not used.	Information on the topic is inaccurate or sparse. The response reports facts.	No response is supplied or the information on the topic is incorrect.
